# Which factors engage women in deprived neighbourhoods to participate in exercise referral schemes?

**DOI:** 10.1186/1471-2458-8-371

**Published:** 2008-10-26

**Authors:** Melanie Schmidt, Saida Absalah, Vera Nierkens, Karien Stronks

**Affiliations:** 1 Academic Medical Centre, University of Amsterdam, Department of Social Medicine, P.O. Box 22660, 1100 DD Amsterdam, The Netherlands

## Abstract

**Background:**

Exercise referral schemes (ERS) have become a popular way of promoting physical activity. The aim of these schemes is to encourage high risk patients to exercise. In evaluating these schemes, little attention has been paid to lower socio-economic groups in a multi-ethnic urban setting. This study aimed to explore the socio-demographic and psychosocial characteristics of female participants in ERS located in deprived neighbourhoods. The second aim was to determine which elements of the intervention make it appealing to participate in the scheme.

**Methods:**

A mixed method approach was utilized, combining a cross-sectional descriptive study and a qualitative component. In the quantitative part of the study, all female participants (n = 523) filled out a registration form containing questions about socio-demographic and psychosocial characteristics. Height and weight were also measured. In the qualitative part of the study, 38 of these 523 participants were interviewed.

**Results:**

The majority of the participants had a migrant background, a low level of education, no paid job and a high body mass index. Although most participants were living sedentary lives, at intake they were quite motivated to start exercising. The ERS appealed to them because of its specific elements: facilitating role of the health professional, supportive environment, financial incentive, supervision and neighbourhood setting.

**Conclusion:**

This study supports the idea that ERS interventions appeal to women from lower socio-economic groups, including ethnic minorities. The ERS seems to meet their contextual, economic and cultural needs. Since the elements that enabled the women to start exercising are specific to this ERS, we should become aware of whether this population continues to exercise after the end of the scheme.

## Background

Many studies have demonstrated the positive relationship between physical activity and health [1,2]. Despite these benefits, a large proportion of adults in European countries fail to achieve the minimum recommendation of 30 minutes of moderately intense physical activity every day [3]. People in lower socio-economic groups meet the current target less frequently than the rest of the population. Within this group, there is an even greater risk among people with a migrant background [4,5]. This is reflected in the results of various studies that show a decreased likelihood of being physically active among residents of deprived neighbourhoods [6,7] and a lower likelihood of using sport facilities for physical activity in deprived areas [8]. This means that major health gains can potentially be achieved in these groups.

Exercise referral schemes (ERS) have become a major way of promoting physical activity among primary care patients, especially in the United Kingdom [9-12]. The aim of these schemes is to encourage and support patients with signs of lifestyle diseases to engage in exercise activities, to become aware of the positive effects of physical activity and to produce changes in behaviour in favour of an active lifestyle [10]. The ERS intervention increases opportunities for exercising by providing access to exercise facilities or activities [9].

ERS have been shown to increase participation in exercise activities in certain populations, at least in the short term [9,13-15]. However, little is known about the applicability of the ERS for lower socio-economic groups and migrant populations in particular. In general, cultural differences and practical problems like language barriers make it difficult to reach these groups in health promotion activities. It has been repeatedly shown that some barriers to physical activity are unique to women from different racial and ethnic backgrounds [16]. For example, Muslim women prefer an all-female environment [17]. We do not yet know whether an ERS is a potentially productive strategy for promoting physical activity among lower socio-economic groups, including ethnic minority groups. A first step in exploring this further is to find out whether the ERS strategy adequately addresses perceived barriers and meets the specific needs and living conditions of these groups.

In this paper we will focus on an ERS in a large city in the Netherlands (The Hague). Since 2002, this intervention has been used to encourage the patients of general practitioners (GPs) living in five deprived neighbourhoods to pursue a more active lifestyle. Patients are referred to a 20-week exercise programme by a GP or other health professional. The study described in this paper aimed to explore the characteristics of the participants and the elements of the intervention that were perceived to be important to their participation in the ERS.

In view of this aim we formulated the following research questions:

1. What are the socio-demographic characteristics of the female participants in this ERS?

2. What are the psychosocial characteristics of the female participants (i.e. attitudes, sense of self-efficacy and social support)?

3. What elements of the intervention make participating in the ERS appealing to the female participants?

### Description of the intervention

The ERS in The Hague is the initiative of the organization STIOM, a foundation that is supportive to health care and social services in the deprived neighbourhoods of The Hague at the request of and in collaboration with GPs and social workers. The participants of the ERS are between the ages of 24 and 55, and the GP or other health professionals believe the health of these patients would benefit from exercise. When the participants contacts the ERS, an appointment is made with a 'sport advisor'. This sport advisor carries out an intake and discusses the different activities, and together they choose which activity suits the client best. This advisor refers the participant to one of the exercise groups: swimming, gymnastics, cardio-fitness or dancing. Participants are expected to pay 11 euro's for a 10-week period of weekly exercise sessions. After 10 sessions, the participants go back to the advisor, where they have the opportunity to purchase a second card for 10 lessons. After the 20 lessons, they return to the advisor to discuss the patient's progress, and receive a certificate. From then on, the participants are expected to have become competent enough to engage in exercise activities on their own.

## Methods

We used a mixed method research design. We used a questionnaire to obtain insight into the socio-demographic and psychosocial characteristics of the participants. In-depth interviews were conducted among a selection of ERS participants to gain a deeper understanding of the elements of the intervention perceived to be important for participation in such an ERS. The study was designed with reference to the research code for qualitative research of the Academic Medical Centre, University of Amsterdam. In line with Dutch legislation, the study was approved by the Netherlands Organization for Health Research and Development and judged to need no further review by a medical ethics committee as participants were recruited on a volunteer basis and were not required to undergo physical examination.

### Questionnaire and registration

#### Participants

This questionnaire was an integral part of the intake and included all female individuals who joined the ERS in The Hague between May 2005 and April 2006 (n = 523). The four largest ethnic groups were made up of Dutch, Turkish, Moroccan and Surinamese participants. Ethnicity was defined as being born in the Netherlands, Turkey, Morocco and Surinam respectively.

#### Questionnaire and data collection

At the time of intake (which took place after the referral), a 'fitness advisor' filled out the questionnaire, which consisted of four types of variables: (1) demographic features (overview in table 1), (2) goals of the participants and (3) physical activity in leisure time. Section 3 contained questions that originated from the Short QUestionnaire to ASsess Health-enhancing physical activity (SQUASH) [18]. The fourth section consisted of 14 statements regarding psychosocial factors (overview in Table 2). These statements originated from a previous study that contained 100 statements [19]. Based on discussions with fitness advisors, 14 statements were selected as the most salient ones. Attitude towards physical exercise was measured using eight items about perceived consequences of exercising. Social support was measured by three items. Self-efficacy expectations were measured by three items about the difficulties participants expected when finding themselves in challenging situations (e.g. bad weather). A 3-point Likert scale was used. Finally, height and weight were measured. Participants were assured their answers would be confidential and that the analyses were anonymous.

**Table 1:** Socio-demographic characteristics of the participant population (n = 523)*

**Country of origin**	**Total**	**The Netherlands (21%) n = 108**	**Morocco (16%) n = 82**	**Turkey (21%) n = 112**	**Surinam (29%) n = 149**	**Other (14%) N = 72**
**Referred by**						

General practitioner	58%	60%	68%	54%	54%	54%

Physiotherapist	23%	15%	21%	35%	26%	11%

Other	20%	25%	11%	12%	20%	35%

**Paid job**						

Yes	24%	30%	11%	13%	36%	20%

No	76%	70%	89%	87%	64%	80%

**Education**						

None	11%	0%	29%	24%	1%	8%

Primary school	36%	19%	34%	55%	36%	38%

Secondary school	14%	20%	9%	6%	22%	8%

Trade school/LBO/VMBO/MULO**	13%	25%	4%	1%	20%	10%

Senior secondary vocational education (MBO)	12%	19%	12%	3%	13%	13%

Higher professional education (HBO)	5%	9%	2%	1%	5%	11%

Other or unknown	8%	7%	10%	11%	5%	13%

length of residence in the Netherlands in years (sd)***	-	-	19.7 (7.8)	19.2 (9.6)	24.1 (9.3)	13.6 (9.8)

**Marital status**						

Married/Living together with partner	54%	40%	83%	73%	35%	48%

Single/Divorced/Widow/Widower	47%	60%	17%	27%	65%	52%

Children, mean (sd)***	1.58 (1.5)	0.8 (1.0)	2.56 (1.6)	2.14 (1.6)	1.22 (1.1)	1.54 (1.5)

**Median age**	45	47	39	42	49	47

BMI, mean (sd)***	31 (7.3)	33 (9.3)	30 (4.8)	33 (8.1)	30 (6.0)	32 (5.8)

**Physical activity during leisure time**						

Walking/Biking/Gardening and doing odd jobs						

0 h/w	24%	18%	20%	21%	31%	30%

0–2 h/w	23%	18%	23%	30%	21%	28%

2–4 h/w	20%	19%	19%	21%	21%	18%

4–6 h/w	9%	10%	13%	9%	8%	4%

> 6 h/w	24%	35%	26%	19%	19%	20%

Gymnastics/Dancing/Swimming/Other sports						

0 h/w	81%	82%	88%	76%	78%	83%

0–2 h/w	14%	10%	11%	19%	15%	11%

2–4 h/w	4%	3%	1%	4%	5%	3%

4–6 h/w	1%	2%	0%	1%	1%	1%

> 6 h/w	1%	4%	0%	0%	0%	1%

* The percentages are rounded numbers.

** ^1 ^LBO = Lower secondary vocational education, MBO = preparatory secondary vocational education, MULO = advanced primary education

*** sd = Standard deviation

**Table 2:** Socio-psychological statement scores of the four major ethnic groups regarding attitude, social support and self-efficacy.

**Means of ethnic group**	**Dutch (21%) Mean (sd)**	**Moroccan (16%) Mean (sd)**	**Turkish (21%) Mean (sd)**	**Surinamese (29%) Mean (sd)**	**Other (14%) Mean (sd)**	Significant differences*
**Attitudes, pro**						

1. If I am physically active on a regular basis, I feel good.	2.65 (.60)	2.65 (.65)	2.60 (.69)	2.76 (.57)	2.75 (.53)	N.S.

3. If I am physically active on a regular basis, I think less about my problems.	2.47 (.73)	2.61 (.65)	2.50 (.73)	2.74 (.55)	2.54 (.74)	D < S

4. If I am physically active on a regular basis, I meet new people.	2.75 (.53)	2.75 (.56)	2.68 (.58)	2.75 (.61)	2.73 (.53)	N.S.

5. An advantage of physical activity is that I am out of the house more often.	2.08 (.92)	2.43 (.81)	2.35 (.81)	2.62 (.72)	2.52 (.72)	D < M, S, O

6. I find it enjoyable to be physically active on a regular basis.	2.57 (.65)	2.84 (.43)	2.72 (.53)	2.86 (.42)	2.74 (.53)	D < M, S

**Attitudes, con**						

2. Exercising is more for men than for women.	- 1.02 (.14)	- 1.20 (.56)	- 1.21 (.57)	- 1.10 (.38)	- 1.15 (.46)	D < M, T

7. If I am physically active, I feel embarrassed.	- 1.50 (.78)	- 1.38 (.66)	- 1.51 (.77)	- 1.25 (.59)	- 1.49 (.72)	D, T > S

8. I am too old to start exercising.	- 1.07 (.32)	- 1.17 (.47)	- 1.24 (.55)	- 1.10 (.41)	- 1.21 (.57)	T > D

**Social support**						

Support from husband/wife	2.49 (.75)	2.13 (.83)	2.04 (87)	2.27 (.89)	2.36 (.78)	T < D

Support from other relatives	2.20 (.84)	2.15 (.81)	2.16 (.81)	2.13 (.83)	2.18 (.78)	N.S.

Support from friends	2.10 (.74)	2.11 (.73)	2.08 (.75)	1.99 (.70)	1.97 (.78)	N.S.

**Self-efficacy (confident they will exercise regularly)**						

During bad weather	2.80 (.52)	2.69 (.68)	2.45 (.80)	2.61 (.70)	2.37 (.84)	D > T, O M > O

When feeling tired	2.35 (.72)	2.10 (.84)	1.93 (.87)	2.17 (.83)	2.13 (.85)	D > T

When feeling stressed	2.65 (.60)	2.42 (.76)	2.34 (.84)	2.54 (.68)	2.51 (.70)	D > T

* N.S. = non-significant, D = Dutch, M = Moroccan, S = Surinamese, T = Turkish, O = Other

#### Analyses

The questionnaires were analysed using SPSS 11.5 software for Windows [20] and descriptive statistics were generated. Differences between the mean scores between ethnic groups in the three psychosocial factors of attitude, social support and self-efficacy were tested with Tukey HSD and Games Howell [21].

### Semi-structured qualitative interviews

#### Participants

To address the third research question regarding the elements that make the ERS appealing, a selection of the 523 participants were invited for in-depth interviews. We used purposive sampling to obtain a cross-section of the population and insight into a potentially broad range of characteristics and personal perspectives. People were invited to participate based on their gender, ethnic background, the type of exercise, their age and the number of lessons followed. With these criteria in mind, participants were approached by researchers visiting the ERS lessons. Participants were recruited for the interviews until new themes stopped emerging from the data. We ended up with 38 interviews. Participants were asked to take part on a voluntary basis and were assured their information would be analysed anonymously. Characteristics of the respondents are shown in Appendix 1 (see additional file 1).

#### Procedure

We interviewed the participants in their homes or at the community health centre using a topic list, which had been developed around the research questions. The topic list was informed by literature and preliminary interviews with sport advisors. These preliminary interviews were conducted to gain insight into how sport advisors perceived the barriers and stimulating factors that play a role for participants in deciding whether to quit or continue with the ERS. The final list covered five topics: (1) personal background, (2) background of the referral, (3) past involvement in physical activity, (4) experience with the ERS and (5) experienced effects. The interviews lasted an average of one hour. The interviews with the Dutch and the Surinamese women were conducted by two researchers (MS and SA). Two bilingual medical students fluent in Berber, Arabic and Turkish, conducted interviews with Moroccan and Turkish participants. The students received thorough training from the researchers. All interviews were taped and transcribed verbatim. Interviews done in Berber, Arabic and Turkish were translated during the transcription process.

#### Analyses

We used an open approach to analyse the interviews to explore the participants' perceptions regarding physical exercise and the elements in the intervention that were perceived to be important for participation in an ERS. Two researchers (MS, SA) coded the same two transcripts independently of each other, and the two versions were then discussed and integrated. Furthermore, one researcher (SA) coded the remaining 36 transcripts and condensed the data by summarizing all the elements the participants mentioned. Transcripts were then categorized to see how widespread the identified themes were in this sample. Categories were added to reflect as many of the nuances in the data as possible. The programme MAXQDA was used to facilitate data coding.

## Results

### Socio-demographic characteristics of the participants

Of the 523 participants, 80% were between 31 and 60 years of age. It was an ethnically mixed group, and included patients who came from 12 different countries. The majority (87%) had a Dutch, Moroccan, Turkish or Surinamese background (Table 1). The mean residence duration for the Turks and Maroccans was around 19 years and for the Surinamese this was about 4 years longer. Of the Turks and Moroccans, 13% and 11% had paid jobs compared to 30% and 36% of the Dutch and Surinamese respectively. The Turks and Moroccans also had a lower level of education: 63% of the Moroccans and 79% of the Turks had had no education or primary school only, compared to 19% of the Dutch and 37% of the Surinamese. There were also differences in marital status: 83% of the Moroccans and 73% of the Turks were married or living together with a partner, compared to 40% of the Dutch and 35% of the Surinamese. The Turks (42) and Moroccans (39) were in general younger than the Dutch (47) and Surinamese (49). Turks and Moroccans generally had more children (2.1 and 2.6 respectively) than the Dutch and Surinamese (0.8 and 1.2 respectively). Finally, almost all participants suffered from obesity irrespective of ethnic background (Table 1).

At the time of intake, 80% to 90% of the participants reported they did not exercise on a health enhancing level. A small proportion mentioned exercising (e.g. swimming or gymnastics) two hours a week or more. Most participants (76%) did report leisure-time physical activity; in particular walking and biking. However, they reported to do these activities at a low to average degree of intensity. Furthermore, the majority of the participants (61%) considered their level of activity to be inadequate.

At the time of intake, most participants in all groups were quite motivated to start exercising. Overall, they had a positive attitude, perceived social support and had a high self-efficacy towards exercising (Table 2). Although there are some significant differences between the ethnic groups (Table 2), the patterns were inconsistent.

Participants reported a moderate level of support from their social environment. There were no ethnic differences in the perception of social support from relatives and friends. However, Turkish (2.04) participants significantly felt less supported by their partners than the Dutch participants (mean 2.49). Most participants appeared to have a moderate to high degree of self-efficacy. The Dutch generally reported higher self-efficacy expectations than the other groups. For example, they have a higher self-efficacy expectations than the Turks when the weather is bad (2.80 and 2.45 respectively) or when they experience stress (2.65 and 2.34 respectively).

### Appealing elements of the intervention

In-depth interviews were used to further explore the elements of the intervention participants considered to be important for joining the ERS. Five characteristics of the intervention emerged and are described below.

#### The Facilitating role of the health professional

About half of the participants highly valued the doctor's advice that they should become physically active. They experienced this advice as being either optional or compulsory. This advice was understood differently by the various ethnic groups. Many Dutch experienced the advice as being 'just a recommendation', which meant it was not experienced as a deciding factor in becoming physical active. It helped them find a place where they felt comfortable exercising. Many migrant participants, however, experienced the GP as someone 'who knows better' and participated in the intervention because they were told to do so.

If the doctor tells me that being overweight is not good for my health and that I have to lose 10 kilos, that's what I'll do. (Turkish woman, age 53, respondent no.24)

Specifically among Turkish and Moroccan participants, the referral's importance concerns the social environment. Some female participants said their partners generally did not allow them to leave the house, but that they allowed them to participate in the ERS because of the significance of the GP's opinion that the women needed to exercise for health reasons. So for these women, the referral appeared to have had the additional effect of providing a legitimate reason to exercise.

#### Supportive environment

There were strong feelings of shame about body weight among the participants, and in particular among overweight Dutch women. Some participants mentioned they did not dare go to a sport school because the women who exercise there are all slim. Other participants see the small groups in this ERS intervention as a step towards daring to enter bigger fitness centres.

Yes, because I'm not going to jump around among all those slim people. (Dutch woman, age 52, respondent no. 3)

Feelings of reluctance also originate from religious and cultural principles. Muslim women in particular mentioned feeling embarrassed in mixed-sex groups due to their cultural and religious backgrounds. Some of them even refused to participate in fitness lessons when the instructor was male or when there were male participants. Some ethnic Dutch women also said they preferred female groups to mixed ones 'just because it feels better'. Some mentioned being ashamed of their weight or physical condition as important factors. For both groups of women, being in a mixed group would mean they would feel uncomfortable about their clothing in front of men, including a male instructor.

Normally I wear a headscarf. If there were men present I'd have to wear a headscarf, now I don't have to. You don't have to wear a headscarf and you're among women and because of this you feel comfortable. (Turkish woman, age 22, respondent no. 27)

In addition to the small groups and their single-sex nature, in order to feel safe, a third element in the intervention seems to be important to the participants: they mentioned that they consider the ERS to be ideal because it gives them the opportunity to exercise with other people who have health problems. Some said they found it encouraging that the group was made up of friendly participants with similar health conditions, and this is also mentioned as a stimulus for continuing to exercise: 'If she can do it, maybe I can too.'

#### Financial incentive

Participants paid 22 euro's for the 20-week period of weekly exercise sessions. The majority of the participants perceived these costs as a difficulty. Nevertheless, they were prepared to pay these costs in order to improve their health. Some found paying for a sport facility to be self-evident, whereas others thought it should be paid for by their health insurance because they had been referred by their GP. Although the ERS was affordable for many participants, they considered the costs of regular sport facilities to be too high and as a barrier to continuing to exercise after the ERS. For half of them, it was actually a reason to stop exercising.

Well, I was enthusiastic about this, because I anyway wanted to start exercising. But a fitness centre will cost you around 45, 55, 65 euros, and I can't pay that. I'll be honest, we have a low income. I don't have a job, my husband doesn't have a job and because of that you have to get by with little things. So for me, this intervention was a way to do this. (Turkish woman, age 50+, respondent no. 25)

#### Supervision by fitness instructors

Supervision by a professional instructor was often given as a reason to participate. Some participants considered supervision to be of major importance in light of their health complaints. Some participants even think of the ERS as a medical project because of the GP's involvement and because they were referred on account of their poor health. In general, participants stressed the stimulus provided by the presence of the fitness instructor, who can give advice when necessary.

...other fitness centres don't have this – they don't have time for people who come there exercise. Although they do explain a little that you have to do this 10 times and so forth, they don't have time to talk to you about it. But people with chronic pain need this. This is what I think anyway – I can't speak for others, of course, but I find it helpful. (Surinamese woman, age unknown, respondent no.16)

#### Neighbourhood setting

The neighbourhood setting was mentioned in two ways. First, for many participants, the distance to the sport facility is an important factor. They prefer a facility in the neighbourhood so they can walk there. The Turkish and Moroccan women in particular said they were not familiar with public transportation and preferred to walk to the facility. Also, although some people who own a car do not feel put off by having to travel some distance, others are motivated by a facility in their own neighbourhood, irrespective of having a car.

Secondly, the neighbourhood setting was given as a reason for the unpopularity of sport activities in the evening among most of the women. Participants do not feel safe in their neighbourhoods, and for some this is a reason to stay at home. Participants who do go out are accompanied by a partner or a group of neighbourhood women.

I did not like the neighbourhood. The first time I came here I was so scared...I had to get off the tram by the market, but I got out at the wrong stop and had to walk along streets with all those men...I was scared and thought, what should I do? ...(Dutch women, age 59, respondent no.14)

Additionaly, Moroccan and Turkish women mentioned being anxious about potential gossip within their social community. Furthermore, Muslim participants would rather stay at home in the evening to fulfil their religious commitments.

We keep an eye on each other, we Moroccans...you know I'm always scared, if we are going at 21.00 o'clock and someone sees me he/she is going to wonder...I don't want others to talk about me... (Moroccan woman, age unknown, respondent no. 34)

## Discussion

A growing amount of literature shows the extent of use and effects of ERS, however, considerably less is known about participant characteristics of the ERS and the participation of ethnic minorities in these schemes. Our findings show that the ERS is able to attract people from lower socio-economic groups, supporting the evidence of Gidlows recent study that residents of deprived neighbourhoods are reached [22]. Additionally, this ERS seems to be able to attract migrant women who in general experience a poorer health than the ethnic Dutch [23]. This is important when considered in the light of the work of Harrison who showed that ERS are unlikely to contribute to population levels of physical activity for they appear to reach only a small percentage of the sedentary population [10]. He suggests that the ERS might be best reserved for those who are most in need of supervised exercise. This group of migrants might be considered one of those specific groups since this intervention seems to meet their specific needs by (1) the facilitating role of the health professional, (2) offering a supportive environment, (3) the financial incentive (4) supervision by fitness instructors and (5) the neighbourhood setting.

A limitation of this study is that we have no information on those who do not enrol and therefore it is not possible to establish whether or not the participants differ from the general population that was referred. The population studied consisted of merely patients who were referred and showed up for an intake. Given the high scores on the psychosocial factors, this population seems to be a selective group of individuals who are already motivated to start exercising. However, although these respondents can be considered motivated, they were living sedentary lives. For this matter it is interesting to understand why they do start exercising within this ERS. Our study does present useful insights into the appeal of these schemes for this specific lower socio-economic group and in particular to ethnic minorities. Future studies should include non-participants as well to see whether our population is a selected group.

In line with other studies of GP exercise schemes [24], we have found that participants considered a medical recommendation to be important to their participation. However, with regard to actually providing access to an exercise facility, in these non-Western migrant populations, the health professional seemed important not only as a motivator but as a facilitator as well, serving as a means to overcome several barriers by providing a supportive environment in which individuals feel comfortable.

The women in this ERS experienced the group activities as supportive, especially when the group consisted of participants with similar health conditions. This relates to the findings in other studies showing that having someone to exercise with would increase women's motivation to engage in the activity [25,26] especially when they are referred as well [27]. This accommodates the opportunity to socialise which is considered important. More in general, social support has shown to be a strong predictor for physical activity in minority women [28]. The referral and the group dynamics during the lessons in the ERS appeared to accommodate the feelings of social support from instructors and fellow participants and could for some in this group be understood as replacement for the low perceived social support from family and friends.

A neighbourhood setting was also one of the elements that appealed to the respondents. Other studies have also devoted attention to the proximity to sport facilities [6,8]. However, the existence of facilities for physical activity does not necessarily imply that the facilities will be used, especially by lower socio-economic groups, since it costs money to use them. The ERS are a way to guide motivated individuals to the regular exercise facilities by providing access to them. However, since aforementioned factors that appeal to the respondents are specific to the ERS (referral, supportive environment, supervision etc) it is not self-evident that the participants will continue exercising on their own in regular facilities, especially since the costs of regular facilities are in general much higher than the own contribution of the ERS.

## Conclusion

In short, this study supports the idea that ERS interventions can appeal to women from lower socio-economic groups in a multi-ethnic urban setting. At the time of intake most participants were aware of the positive effects of physical activity on health, and in terms of attitude, social support and self-efficacy they were also quite motivated. Even so, they were living sedentary lives. This suggests that this population needs more than being motivated in order to become physically active. This ERS seems to meet their specific needs. Moreover, this study has shown that specific elements appeal to them: (1) the facilitating role of the health professional, (2) supportive environment, (3) financial incentive, (4) supervision by fitness instructors and (5) the neighbourhood setting.

For these lower socio-economic and ethnic minority groups, it is possible that when they complete the programme, the elements of the ERS that appealed to them might reappear as barriers. Effective interventions require adequate resources to support the duration needed for physical activity behavioural change [29] This might imply that follow-up projects are needed to help participants find activities suitable to their socio-economic and socio-cultural characteristics.

## Competing interests

The authors declare that they have no competing interests.

## Authors' contributions

MS and KS wrote the original study proposal. KS, SA, VN, MS contributed to the design of the study. SA and MS coordinated and carried out data collection and analyses. KS and VN supervised all data collection, analyses and interpretation. All authors contributed to the drafting of the article and approved the final manuscript.

## Pre-publication history

The pre-publication history for this paper can be accessed here:



## Supplementary Material

Additional file 1 **Profile of the interviewed participants.** This table provides the characteristics of the interviewed participants. Click here for file
