# Supplementary material for: Which factors engage women in deprived neighbourhoods to participate in exercise referral schemes?
Source: BMC Public Health. 2008 Oct 26;8:371. doi: 10.1186/1471-2458-8-371 (PMC2583997; doi:10.1186/1471-2458-8-371)
Supplement: Additional file 1 — Profile of the interviewed participants. This table provides the characteristics of the interviewed participants. [file 1471-2458-8-371-S1.doc]

Appendix 1. Profile of interviewed participants.

| **No.** | **Country of birth** | **Age** | **Type of exercise** | **Gender** |
| --- | --- | --- | --- | --- |
| 1 | Netherlands | 51 | Swimming | F |
| 2 | Netherlands | Unknown | Fitness training | F |
| 3 | Netherlands | 52 | Fitness training | F |
| 4 | Netherlands | 59 | Dancing | F |
| 5 | Netherlands | 25 | Fitness training | F |
| 6 | Netherlands | 55 | Fitness training | F |
| 7 | Netherlands | 55 | Fitness training | F |
| 8 | Netherlands | 45 | Fitness training | F |
| 9 | Netherlands | 61 | Fitness training | F |
| 10 | Netherlands | 55 | Fitness training | F |
| 11 | Surinam | 58 | Dancing | F |
| 12 | Surinam | 57 | Swimming | F |
| 13 | Surinam | 58 | Swimming | F |
| 14 | Surinam | Unknown | Swimming | F |
| 15 | Surinam | 58 | Swimming | F |
| 16 | Surinam | Unknown | Fitness training | F |
| 17 | Surinam | Unknown | Fitness training | F |
| 18 | Surinam | Unknown | Swimming | F |
| 19 | Surinam | 32 | Fitness training | F |
| 20 | Turkey | 36 | Fitness training | F |
| 21 | Turkey | 45 | Swimming | F |
| 22 | Turkey | 31 | Swimming | F |
| 23 | Turkey | Unknown | Fitness training | F |
| 24 | Turkey | 53 | Swimming | F |
| 25 | Turkey | 50+ | Swimming | F |
| 26 | Turkey | 45 | Fitness training | F |
| 27 | Turkey | 22 | Dancing | F |
| 28 | Turkey | 25 | Fitness training | F |
| 29 | Morocco | 52 | Fitness training | F |
| 30 | Morocco | 28 | Dancing | F |
| 31 | Morocco | 50+ | Dancing | F |
| 32 | Morocco | 36 | Fitness training | F |
| 33 | Morocco | Unknown | Swimming | F |
| 34 | Morocco | Unknown | Swimming | F |
| 35 | Morocco | 22 | Fitness training | F |
| 36 | Morocco | 30 | Swimming | F |
| 37 | Morocco | 32 | Dancing | F |
| 38 | Tunisia | 37 | Fitness training | F |
